# Supplementary material for: Meta-analysis of QTLs associated with popping traits in maize (Zea mays L.)
Source: PLoS One. 2021 Aug 19;16(8):e0256389. doi: 10.1371/journal.pone.0256389 (PMC8376040; doi:10.1371/journal.pone.0256389)
Supplement: Attachment — Submitted filename: S Response to reviewer.docx [file pone.0256389.s004.docx]

Journal requirement:

| **Sl. No.** | **Comments** | **Response** |
| --- | --- | --- |
| 1. | Please ensure that your manuscript meets PLOS ONE's style requirements, including those for file naming | The article has been formatted as per the PlosOne style requirement. |
| 2. | In your Methods section, please ensure you have included all the details of the search strategy for literature review, *i.e.* search keywords/date/language, inclusion/exclusion criteria, etc. for reproducibility purpose | In the material and method section the keywords used to search the research/review articles and the search engines used has been added as follows “Published works on QTL mapping for popping traits in maize were identified through database search using the keywords, *viz*., popcorn, QTL, metaQTL, popping expansion volume, popping volume, and surveyed in google search engine and PubMed”. |
| 3. | Thank you for stating the following in the Acknowledgments Section of your manuscript:  "The work was carried out under the financial assistance from the Indian Council of Agricultural Research, New Delhi (India). Inputs of Dr. Shelly Parveen, Indian Agricultural Research Institute, New Delhi (India) for discussion on biochemical interpretation. The first author thanks Punjab Agricultural University for the logistic support during course of investigation."  We note that you have provided funding information that is not currently declared in your Funding Statement. However,  funding information should not appear in the Acknowledgments section or other areas of your manuscript. We will only publish  funding information present in the Funding Statement section of the online submission form.  Please remove any funding-related text from the manuscript and let us know how you would like to update your Funding  Statement. Currently, your Funding Statement reads as follows:  "The author(s) received no specific funding for this work." | The mentioning of funding information has been removed from acknowledgment section in the manuscript.  It is true that for this work we did not receive any specific funding but the general budget from the Indian Council of Agricultural Research (ICAR) to support research activities was key in carrying out this work. We would like to acknowledge ICAR in some form or other. We may be guided how may we put it. |

Comments of Reviewers:

Reviewer #1: Minor comments:

| Sl. No. | Comments | Response |
| --- | --- | --- |
| 1. | Abbreviations should be explained in their very first appearance in the text, please explain these abbreviations: QTL (line 9), CGAR (line 28), psi (line 39), PEV (line 54), DAP (line 320), and describe what is B73 (line 329). | All the abbreviations have been incorporated in the text in the desired positions:  Quantitative trait locus (QTL),Compound Annual Growth Rate (CAGR), pounds per square inch (psi), Days after pollination (DAP)*, Popping Expansion Volume (PEV), B73 (the first fully sequenced maize reference genome)  *DAP has been mentioned earlier also (line number 222 and the abbreviation is inserted there itself) |
| 2 | Line 180, there should be space in “tenmetaQTL” | Space has been inserted as mentioned |
| 3 | Line 206-271: As describing the “The features of the four major metaQTLs identified in the study” no need of describing the features of the metaQTLs that already mentioned in the Table 3. Most of the features already mentioned in Table 3. | The repeated information mentioned in the text have been removed and only the information about the genes identified in the metaQTL region with their expression pattern are retained. |
| 4 | You could add a supplementary table describing the features of the individual QTL from different studies that used in this article. | One supplementary table has been added (Table S2: Features of the individual QTL related to popping traits in maize reported in different studies) describing the name of QTL, position in the map, confidence interval and R^2^ value for each QTL. |

Reviewer #2: Summary:

| Sl. No. | Comments | Response |
| --- | --- | --- |
| 1. | Only papers published between 2006 and 2012 are included for the metaQTL analysis. It is not clear why you did not included papers after 2012. At lease, I find one paper in 2021 "Thakur, S., Kumar, R., Vikal, Y. et al. Molecular mapping of popping volume QTL in popcorn (Zea maize L.). J. Plant Biochem. Biotechnol. (2021). https://doi.org/10.1007/s13562-020-  00636-y". I think it needs some justification for not including papers published after 2012. | The paper, *viz*., “Thakur S, Kumar R, Vikal Y et al (2021) Molecular mapping of popping volume QTL in popcorn (Zea maize L.). J. Plant Biochem. Biotechnol.<https://doi.org/10.1007/s13562-020-00636-y>". has been published online on January 11, 2021 but our study has been conducted much before that and the initial submission in plosOne was done on December 4, 2020. In literature total 10 articles were available, out of ten reports two could not be used because the used primers were coded (instead of marker names) (Lu et al. 2003), in addition SNP markers were used (Poster and Thomas 2015) to construct the maps which could not be plotted to the consensus SSR map because of uncommonness of markers. Except these two articles, all the available studies were included. |
| 2 | There is inconsistency in citing references in text. You used both "number" and "author-date" styles. Your citations in the text need to be consistent as recommended by the journal. | All the references have been formatted in the PlosOne reference style. |
| 3 | The Figures are poor in quality and I think they need to be improved in the final submission. | The pixels of the images have been enhanced for Figure 2 and Figure 3. |
| 4 | I think it is good to avoid using "etc" in listing things (example on line 19: "MetaQTL1_1 at bin location 1.01 coincided with the reported QTLs related to various agronomic traits like stalk diameter, tassel length etc."). If the list is not big enough, I think it is better to mention all of them. This works for the other "etc" mentioned in the text. | The name of all the traits (stalk diameter, tassel length, leaf area and plant height) have been mentioned and etc. has been removed.  Further, seed weight, yield, disease resistance etc. has been replaced as seed weight, yield, disease resistance, insect resistance and stalk strength in line number 74. |
| 5 | Abbreviations must be defined first before they can be used the next time it comes in the text. (examples: "PEV" on line 54 and "PVE" on line 81). | All the abbreviations have been defined in the first place as also pointed out by the reviewer 1. This has been pointed out by Reviewer 1 as well and the issue has been addressed. |
| 6 | The discussion part: The first part (paragraphs 1 and 2) is discussed in comparison with past research or generally describing concept or facts referring to literature. I think this is good. The second part (paragraph 3) seems a repetition of the results part. I suggest you look into the discussion part and try to improve it. | The suggestion has been addressed. The paragraph 3 describes the genes identified in the metaQTL regions from the reference genome and most of the study have not reported the metaQTL at gene level. But we have identified one article which describes the genes linked to SNPs for popping expansion (Senhorinho et al 2019) and it has been discussed in the article as per the suggestion. |
| 7 | Line 204-206: You declared 4 metaQTLs important because they were represented by 5-8 QTLs. Is this enough to classify some as important and the others as not important. I have not seen any other criteria for your decision to classify as important and not important. I think this needs some explanation. I think some other criteria should be added in addition to the number of QTLs. This simple classification may sideline some of the metaQTLs which can be important in breeding. | Thank you for the suggestion. More logic has been added for selecting the four metaQTLs out of the 10 identified metaQTLs which states that “The four major metaQTLs namely metaQTL1_1, metaQTL1_5, metaQTL1_7 and metaQTL6_2 are considered important for popping traits as 5-8 QTLs were clustered in these regions with moderately high R^2^ value. The clustered QTLs were also from two (metaQTL1_1 and metaQTL1_5) and more than two (metaQTL1_7: 3 and metaQTL6_2: 4) different experiments. Further, out of the 19 genes specifically involved in carbohydrate metabolism, eleven are residing in these regions”. |
