# Supplementary material for: Meta-analysis of QTLs associated with popping traits in maize (Zea mays L.)
Source: PLoS One. 2021 Aug 19;16(8):e0256389. doi: 10.1371/journal.pone.0256389 (PMC8376040; doi:10.1371/journal.pone.0256389)
Supplement: Attachment — Submitted filename: Response to Reviewers.docx [file pone.0256389.s005.docx]

**Response to reviewers**

**Journal Requirements:**

**Query:** Please review your reference list to ensure that it is complete and correct. If you have cited papers that have been retracted, please include the rationale for doing so in the manuscript text, or remove these references and replace them with relevant current references. Any changes to the reference list should be mentioned in the rebuttal letter that accompanies your revised manuscript. If you need to cite a retracted article, indicate the article’s retracted status in the References list and also include a citation and full reference for the retraction notice.

***Response:*** *The references have been formatted as per the journal requirement and the modifications are highlighted in the in the manuscript with track change mode.*

**Comments of reviewers:**

| **Reviewer #1:** | |
| --- | --- |
| Line 117:” Lod value” Lod should be capitalized. | Lod has been capitalized in Line number 122 (earlier 117) |
| Line 128, table 2: Row 2 and 3, in Reference column, there is no need to add semicolon after references. row 5 in reference column”) [7, 24]”, there is a redundant parenthesis. Also, at the footnote of table 2 describe what is RIL, BC and F mapping population. | Semicolon in reference column at row number 2 and 3 has been removed.  The parenthesis at row number 4 has been removed.  The abbreviation of RIL, BC and F mapping population has been described as footnote in Table 2. |
| Line 185: write the reference of the “q teller”. | Reference of the “q teller” has been added in the text. |
| Line 124, and 210: Fig 2 and 3: All the caption of figures should be Bold. | All the captions in Figure number 2 and 3 has been made in bold. |
| Line 78, 128 and 215: Table, 1,2, 3 captions font are not consistent with the figures caption font, they are smaller. | The font size of Table, 1,2, 3 captions have been changed to 11 (Times new roman) similar to font size of the Figures. |
| Line 312: There is a redundant parenthesis at the end of sentences.”Features of the individual QTL related to popping traits in maize reported in different studies)” | The parenthesis at the end of sentences” Features of the individual QTL related to popping traits in maize reported in different studies” has been removed. |
| General comment: Fig 2 and Fig 3 do not have good quality and resolution. | The quality and resolution of Fig 2 and Fig 3 has been improved as per requirement of PlosOne (https://pacev2.apexcovantage). |
| **Reviewer #2:** | |
| It seems rephrasing sentences necessary in several places in the manuscript:  Example1--- Line 16-17: The clustered QTLs were from ≥2 different experiments and out of the 19 genes specifically involved in carbohydrate metabolism, eleven were in these regions thus considered as most important.  Could be re-written as: The clustered QTLs were from two or more experiments. Of the 19 genes specifically involved in carbohydrate metabolism, 11 of them were in these regions, implying the importance of these clustered QTLs. | Thank you for the observation. The sentence has been reparsed as per the suggestion and changed as “The clustered QTLs were from two or more experiments. Of the 19 genes specifically involved in carbohydrate metabolism, 11 of them were in these regions, implying the importance of these clustered QTLs.” |
| Example2--- Line 18-19: A total of 229 genes were shortlisted in these regions on the basis of their expression in endosperm and pericarp tissues using qteller option in maizeGDB, out of which 19 were specifically involved in carbohydrate metabolism.  Could be re-written as: Based on the expression pattern in endosperm and pericarp tissues, a total of 229 genes were selected. Nineteen of these genes involves in carbohydrate metabolism. | The sentence has been reparsed as “Based on the expression pattern in endosperm and pericarp tissues, a total of 229 genes were selected. Nineteen of these genes involves in carbohydrate metabolism” As per suggestion. |
| In general, it may be good to go through the manuscript and make changes as required. | We have gone through the manuscript as suggested and some changes in the sentences/text has been done and highlighted also. |

**Journal requirement:**

**While revising your submission, please upload your figure files to the Preflight Analysis and Conversion Engine (PACE) digital diagnostic tool, https://pacev2.apexcovantage. com/. PACE.**

The Figures were uploaded to Preflight Analysis and Conversion Engine (PACE) digital diagnostic tool, https://pacev2.apexcovantage. com/. PACE and specifications of the Figures were changed as per the requirement.
