# Supplementary material for: Meta-analysis of QTLs associated with popping traits in maize (Zea mays L.)
Source: PLoS One. 2021 Aug 19;16(8):e0256389. doi: 10.1371/journal.pone.0256389 (PMC8376040; doi:10.1371/journal.pone.0256389)
Supplement: Attachment — Submitted filename: Response to Reviewers.docx [file pone.0256389.s006.docx]

**Response to reviewers**

**Journal Requirements:**

**Query:** Please review your reference list to ensure that it is complete and correct. If you have cited papers that have been retracted, please include the rationale for doing so in the manuscript text, or remove these references and replace them with relevant current references. Any changes to the reference list should be mentioned in the rebuttal letter that accompanies your revised manuscript. If you need to cite a retracted article, indicate the article’s retracted status in the References list and also include a citation and full reference for the retraction notice.

***Response:*** *The references have been formatted as per the journal requirement and the modifications are highlighted in the in the manuscript with track change mode.*

**Comments of reviewers:**

| **Reviewer #1:** | |
| --- | --- |
| 1- The Fig 2 and 3 still do not have good quality and resolution. In Fig 3, name of the markers should be clear. | Figure 2 and 3 have been further enhanced as per the suggestion. |
| 2- Make sure that Table 3 is in good shape:  a) It has one redundant row at the bottom, that should be deleted,  b) Choose brief header, and make the font of the headers and the content of the Table smaller (for example 7) until they can be fit in on or two lines,  c) All digits in Table 3 with decimal should be rounded to 1 or 2 decimal places, be consistent all over the digits.  d) Explain what chr, CI, R2, AIC, kb, and cM are at the footnote of Table. | 2. Table 3 has been formatted in a good shape  a) The redundant row at the bottom is removed  b) Brief header is given with font 8.  c) Digits in the table has been uniformly kept at 2 decimals.  d) Chr, CI, R2, AIC, kb, and cM have been explained at the footnote of Table. |

**Journal requirement:**

**While revising your submission, please upload your figure files to the Preflight Analysis and Conversion Engine (PACE) digital diagnostic tool, https://pacev2.apexcovantage. com/. PACE.**

The Figures were uploaded to Preflight Analysis and Conversion Engine (PACE) digital diagnostic tool, https://pacev2.apexcovantage. com/. PACE and specifications of the Figures were changed as per the requirement.
